# Work2Prevent, an Employment Intervention Program as HIV Prevention for Young Men Who Have Sex With Men and Transgender Youth of Color (Phase 3): Protocol for a Single-Arm Community-Based Trial to Assess Feasibility and Acceptability in a Real-World Setting

**DOI:** 10.2196/18051

**Published:** 2020-09-11

**Authors:** Brandon J Hill, Darnell N Motley, Kris Rosentel, Alicia VandeVusse, Robert Garofalo, Lisa M Kuhns, Michele D Kipke, Sari Reisner, Betty Rupp, Rachel West Goolsby, Micah McCumber, Laura Renshaw, John A Schneider

**Affiliations:** 1 Planned Parenthood Great Plains Overland Park, KS United States; 2 Center for Interdisciplinary Inquiry and Innovation in Sexual and Reproductive Health Department of Obstetrics and Gynecology University of Chicago Chicago, IL United States; 3 Guttmacher Institute New York, NY United States; 4 Division of Adolescent Medicine, Ann & Robert H Lurie Children’s Hospital Department of Pediatrics, Feinberg School of Medicine Northwestern University Chicago, IL United States; 5 Division of Research on Children, Youth, and Families Children's Hospital Los Angeles Los Angeles, CA United States; 6 Fenway Health The Fenway Institute Boston, MA United States; 7 Collaborative Studies Coordinating Center Department of Biostatistics Gillings School of Global Public Health, University of North Carolina at Chapel Hill Chapel Hill, NC United States; 8 Department of Medicine University of Chicago Chicago, IL United States

**Keywords:** HIV/AIDS, youth, young men who have sex with men, YMSM, young transgender women, YTW, gender nonconforming youth, LGBTQ, unemployment, homelessness, sex work

## Abstract

**Background:**

In the United States, young cisgender men who have sex with men (YMSM), young transgender women (YTW), and gender nonconforming (GNC) youth face elevated rates of HIV infection. However, racial and ethnic disparities in adolescent HIV infection cannot be attributed to individual-level factors alone and are situated within larger social and structural contexts that marginalize and predispose sexual and gender minority youth of color to HIV. Addressing broader ecological factors that drive transmission requires interventions that focus on the distal drivers of HIV infection, including violence exposure, housing, food insecurity, educational attainment, and employment. Given the ways that economic instability may make YMSM, YTW, and GNC youth of color vulnerable to HIV exposure, this study focuses on employment as an HIV prevention intervention. More specifically, the intervention, called Work2Prevent (W2P), targets economic stability through job readiness and employment as a means of preventing behaviors and factors associated with adolescent and young adult HIV, such as transactional sex work and homelessness. The intervention was adapted from iFOUR, an evidence-based employment program for HIV-positive adults in phase 1 of this study, and pilot tested in a university-based setting in phase 2.

**Objective:**

This paper aims to describe the protocol for the community-based test phase of W2P. The purpose of this phase was to pilot test a tailored, theoretically informed employment intervention program among YMSM, YTW, and GNC youth of color within a lesbian, gay, bisexual, transgender, and queer (LGBTQ) community setting.

**Methods:**

The employment intervention was pilot tested using a single-arm pretest-posttest trial design implemented among a sample of vulnerable YMSM, YTW, and GNC youth of color using services within a community-based LGBTQ center. Assessments will examine intervention feasibility, acceptability, and preliminary estimates of efficacy.

**Results:**

Phase 3 of W2P research activities began in May 2019 and was completed in December 2019. Overall, 41 participants were enrolled in the community-based pilot.

**Conclusions:**

This study will assess intervention feasibility and acceptability in the target populations and determine preliminary efficacy of the intervention to increase employment and reduce vulnerability to HIV when implemented in a community-based setting serving LGBTQ youth of color. Testing the intervention in a community setting is an opportunity to evaluate how recruitment, retention, and other outcomes are impacted by delivery in a venue akin to where this intervention could eventually be used by nonresearchers. If W2P demonstrates feasibility and acceptability, a larger multisite trial implemented in multiple community settings serving YMSM, YTW, and GNC youth of color is planned.

**Trial Registration:**

ClinicalTrials.gov NCT03313310; https://clinicaltrials.gov/ct2/show/NCT03313310

**International Registered Report Identifier (IRRID):**

DERR1-10.2196/18051

## Introduction

### Background

In the United States, youth of color assigned male at birth who engage in sexual contact with individuals assigned male at birth, including young cisgender men who have sex with men (YMSM), young transgender women (YTW), and gender nonconforming (GNC) youth, face elevated rates of HIV infection [1,2]. In 2018, an estimated 26% of all new HIV diagnoses were among Black men who have sex with men (MSM), while 20% were among Hispanic/Latino MSM [1]. These outcomes are stark, given that Black and Latino individuals comprise 13% and 18% of the US population, respectively [3]. Further, although the Centers for Disease Control and Prevention (CDC) does not provide estimates of HIV infection for transgender and GNC populations due to gaps in public health department data, a previous meta-analysis of US studies found an average HIV prevalence rate of 28% among transgender women, with a higher rate of 56% among Black transgender women specifically [4]. This racial disparity is not solely driven by individual sexual behaviors, but rather a number of interrelated socioecological and structural factors that proximate YTW, GNC youth, and YMSM of color to HIV exposure and disease susceptibility [2,5-11]. In particular, these groups face co-occurring and interconnecting disparities in housing, health care access, social service availability, education, poverty, employment, and violence [12-21]. Economic factors may be especially salient among these populations, as prior research suggests that sexual and gender minority youth of color face hiring bias, job discrimination, low pay, and limited benefits when navigating employment [18,22]. These experiences contribute to a large proportion of sexual and gender minority youth of color living in poverty [18,22-24].

Additionally, economic marginalization may increase potential for HIV exposure and infection by contributing to reliance on survival sex work, or exchanging sex for money, food, shelter, drugs, or other commodities [25-27]. Survival sex work has also been associated with coping-related substance use [12,13,17,20,21]. Previous studies have found that engagement with survival sex work among YMSM, YTW, and GNC of color is also associated with structural factors, including financial insecurity and socioeconomic disconnection [28-30]. Further, engagement in survival sex work may increase youths’ susceptibility to sexually transmitted infections (STIs) and HIV infection by exposing them to sexual networks with higher STI and HIV prevalence, increasing their numbers of sexual partners, and creating challenges in condom use negotiation [28-30].

Given the role that economic instability may play in driving HIV susceptibility among YMSM, YTW, and GNC youth of color, structural interventions are needed in order to address HIV inequities at their root cause [31]. Structural-level interventions target underlying social drivers of poor health and promote agency among marginalized groups in order to facilitate health-positive actions that can benefit both the individual and community [31]. More specifically to HIV, structural interventions, including comprehensive sex education, community- and venue-based HIV/STI testing, stable housing programs, increased health care coverage, and needle exchange programs, have greatly reduced HIV infection [32-34]. However, fewer structural interventions that address distal drivers of HIV infection, including decreased criminalization and poverty and increased economic stability, have been developed and implemented, although these factors have the potential to mitigate broader disparities in HIV [33,34]. Accordingly, employment as HIV prevention has the potential to be a scalable intervention that targets the economic drivers of HIV infection among YMSM, YTW, and GNC youth.

### Rationale for Employment as HIV Prevention

In a recent Chicago-based social network intervention for Black YMSM and YTW aged 18 to 35 years, roughly 45% of participants from the respondent-driven sample reported being unemployed [35]. Across Adolescent Medical Trials Network for HIV/AIDS Interventions (ATN) sites in 17 US cities, 64.6% of adolescents and young adults reported being unemployed, despite 67.8% having completed high school and being a mean working age of 20.4 years [36]. Thus, many youths vulnerable to HIV may benefit from a tailored intervention that specifically addresses job preparedness and workforce engagement [18,22,37]. Additionally, employment interventions may also need to specifically address the experiences of overt and implicit discrimination and mistreatment that sexual and gender minority youth report while job seeking and within the workplace [38-40].

The objective of the Work2Prevent (W2P) study is to adapt and pilot test Increased Individual Income and Independence (iFOUR), an effective, theoretically-driven employment program for HIV-positive adults [41-44], to the needs of vulnerable YMSM, YTW, and GNC youth of color aged 16 to 24 years. For a full rationale of the overall W2P study, please refer to the W2P phase 2 paper [45].

### Theoretical Framework

The W2P study is informed by frameworks from the health belief model [46] and positive youth development [47-49]. More specifically, the W2P intervention is intended to help participants identify barriers to obtaining employment and increase positive beliefs regarding the perceived benefits of employment. This is supported by helping young people understand, value, and develop both external and internal assets that will benefit their pursuit and maintenance of stable, formal employment. For a more thorough explanation of the theoretical framework, please refer to the W2P phase 2 paper [45].

### Rationale for Testing the Intervention in a Community Setting

Phase 3 of the W2P study, the topic of this current paper, focuses on pilot testing the intervention in a community-based social service setting. Implementation science highlights the importance of implementing and evaluating interventions in community settings that more closely reflect the real conditions under which the intervention will be delivered upon being scaled [50-53], given that interventions may perform differently when translated from a university research setting to a community-based organization or clinic [54-56]. Further, community-based interventions are increasingly recognized as essential to achieving the Joint United Nations Programme on HIV/AIDS 90-90-90 targets through their ability to overcome structural barriers to health care access [57]. For example, a community-based implementation of Many Men, Many Voices, a CDC-endorsed evidence-based health intervention for Black MSM, evidenced greater reductions in HIV-related behaviors than in the original randomized trial [58,59]. In translating an intervention from a university setting to a community setting, researchers may identify necessary adaptations and challenges that must be considered to maintain the utility of the intervention. Therefore, in order to ensure that efficacious HIV interventions may be optimally scaled, it is important to implement and evaluate these interventions in community-based clinics and organizations [60].

Assessing how interventions perform in a community setting may be particularly important for interventions that employ a structural-level focus. Indeed, social and structural outcomes are often interrelated. For instance, a previous study of transgender women of color found that employment status was associated with stable housing [61]. Further, the impact of unemployment and job loss on mental health has been well documented [62], often leading to poor mental health outcomes [63] and maladaptive health behaviors [64]. When structural-level interventions are delivered in a university setting, they are generally delivered in isolation and thus do not directly consider how the intervention may interact with services aimed at addressing other related structural factors. However, when an intervention is integrated into an existing service-providing organization, other services that address related structural needs are generally more readily available to study participants. Thus, evaluation of structural-level interventions in community-based settings allows researchers to better assess how intervention performance may be impacted by the social ecology of existing organizations and their corresponding service apparatuses.

Evaluating interventions in community settings, particularly those tailored for marginalized groups, may also be necessary because organizational settings can have an impact on participants’ level of trust and engagement. Due to historical events, like the Tuskegee syphilis study, and negative experiences with the health system [65], many people of color are reluctant to take part in health research [66]. Further, university settings are often unknown to study participants prior to enrollment and thus, initial impressions of the organizations delivering the intervention may be less established. In contrast, delivery of interventions in community organizations and clinics familiar to participants leverages existing relationships in order to make the intervention more reputable to the community. Further, the presence of familiar individuals who vouch for the trustworthiness of the researchers has the potential to decrease mistrust and engender greater participation in an intervention [67]. Understanding how this impacts study participants’ engagement and the performance of the intervention may be critical in ensuring potential scalability. This may be particularly important for interventions targeting sexual and gender minority youth of color, who often express high levels of institutional and medical distrust due to longstanding and historical mistreatment [68,69].

Implementing and evaluating interventions in community settings may also allow researchers to assess how intervention delivery is impacted by real-world logistical concerns [53,70]. In order to implement an intervention in a community setting, it may need to be further tailored to accommodate organizational constraints such as staffing, space, scheduling, technological capacity, and resource supports [53]. Testing an intervention in a community-based organization allows researchers to tailor the intervention to address these real-world constraints and assess how this additional tailoring impacts intervention performance.

## Methods

### Conceptual Model

**Figure 1:**
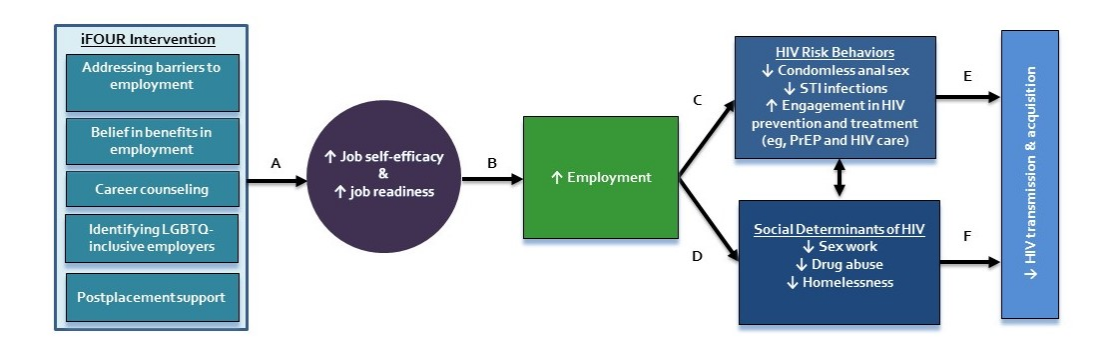
W2P conceptual model. iFOUR: Increased Individual Income and Independence; LGBTQ: lesbian, gay, bisexual, trangender, and queer; PrEP: pre-exposure prophylaxis; STI: sexually transmitted infection; W2P: Work2Prevent.

The W2P conceptual model in Figure 1 draws on the existing iFOUR theoretical framework to hypothesize the potential relationship between adolescent and young adult employment and HIV. The W2P model proposes that employment and subsequent economic connection and stability serve as a structural-level intervention for adolescents and young adults. Our hypothesis is that the adapted and tailored iFOUR intervention will facilitate increased job self-efficacy and job readiness (path A) and ultimately increase employment placement and maintenance (path B). Further, establishing economic stability will decrease engagement in HIV-related behaviors and increase HIV prevention and care (path C), while also decreasing involvement with known social determinants of HIV, such as sex work and substance use (path D), which are directly linked to HIV transmission and acquisition among YMSM, YTW, and GNC youth of color (paths E and F).

### Study Design

W2P uses a mixed methods design. Phase 1 involved the adaptation of relevant intervention components from the existing evidence-based iFOUR employment program for HIV-positive adults [41-43] to YMSM, YTW, and GNC youth of color. Phase 2 involved a pretest of the intervention and study assessments, followed by pilot testing in a university setting to assess feasibility and acceptability and to provide preliminary estimates of efficacy using pretest-posttest comparisons. Phase 3 consists of further refinement of the intervention and study assessments, as well as pilot testing of the intervention in a community-based setting in order to further assess feasibility and acceptability and to provide preliminary estimates of intervention efficacy under real-world conditions using pretest-posttest comparisons. For the purposes of adaptation to a community setting, a number of changes were made between phase 2 and phase 3, including condensing the survey instruments, workshop sessions, and intervention timeline. In phase 2, the intervention workshops were delivered over 2 weeks, and the participants generally completed the baseline survey during a separate study visit within 3 weeks prior to the first intervention session. In phase 3, the intervention workshops were delivered over the course of 2 days, and the first session was offered immediately after participants complete the baseline assessment. In phase 2, participants completed a follow-up visit at 8 months, whereas the follow-up visit was at 3 months for phase 3. For a complete description of phase 2, please refer to the W2P phase 2 protocol paper [45].

### Ethics, Consent, and Institutional Board Approval

W2P has been reviewed and approved by the University of Chicago Institutional Review Board (IRB# 16-1152). Informed consent for this study was obtained in person by study staff before any study-related activities took place. The trial was registered in October 2017 at Clinicaltrials.gov (NCT03313310).

### Participants

Study participants included 41 Black or African American and Hispanic or Latinx YMSM, YTW, and GNC youth. Inclusion criteria included (1) being assigned male at birth, (2) reporting ever having sex with men, (3) identifying as African American or Black or Hispanic or Latinx, (4) aged 16 to 24 years, (5) English speaking, (6) currently unemployed but seeking employment or employed only part-time, defined as working 35 hours or less on average per week, (7) able to attend a 4-session workshop, and (8) did not participate in phase 2 of W2P.

### Study Setting

All study visits were conducted at a community site, the Village. The Village is a community-based setting that provides drop-in services, support groups, resources, behavioral health counseling, community programs, housing and legal assistance, and HIV and STI testing to sexual and gender minority youth. The Village is affiliated with the Chicago Center for HIV Elimination at University of Chicago Medicine and primarily serves Black YMSM, YTW, and GNC youth. This community space is located on the south side of Chicago, a predominantly Black and African American area of the city. A previous spatial study found this area was lacking in career services tailored to the lesbian, gay, bisexual, transgender, and queer (LGBTQ) community [19]. The Village serves approximately 1000 individuals annually, of which 90% are Black or African American, 9% are Hispanic or Latinx, 74% are MSM, 7% are transgender, and 36% are adolescents and young adults aged 24 and younger.

### Recruitment

Planned participant recruitment efforts included recruitment from the community site itself, as well as from primary and community clinics serving YMSM, YTW, and GNC youth, such as Howard Brown Health, a Chicago-based LGBTQ-focused Federally Qualified Health Center, during their youth drop-in programs. Interested participants completed a prescreen survey to assess eligibility. Eligible participants were then scheduled for study visits and workshop sessions.

### Incentives

Study participants were offered compensation for their time. Participants could receive up to US $290 total for complete participation in the form of cash or Visa gift card equivalents. Participants received US $30 for each study visit completed at baseline, postintervention, and 3-month follow-up, up to US $40 for biological specimens at baseline and 3-month follow-up, if provided, and US $30 for each workshop session attended.

### Visit Schedule and Data Collection

**Figure 2:**
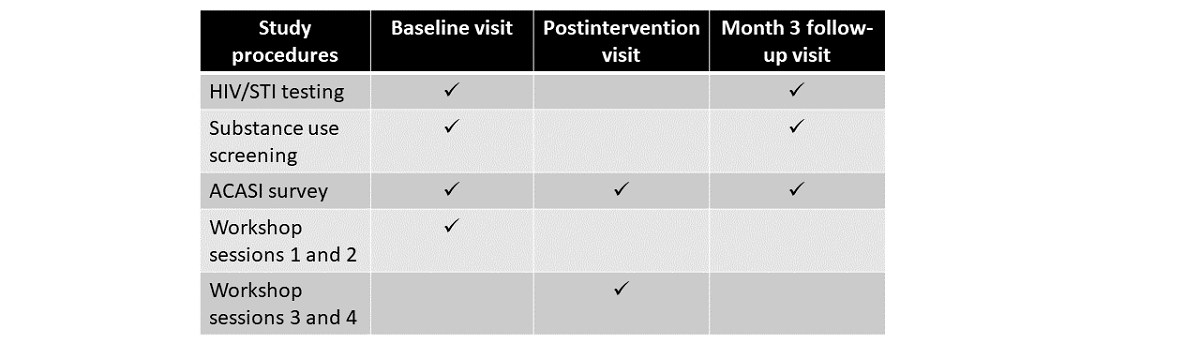
Study procedures. ACASI: audio computer-assisted self-interview.

W2P consisted of data collection across 3 time points, which occurred at baseline, postintervention, and 3-month follow-up, as referenced in Figure 2. A 4-session intervention workshop series occurred over a 2-day period, with workshop sessions 1 and 2 being conducted on the same day as the baseline visit and sessions 3 and 4 on the same day of the postintervention visit.

#### Baseline

Participants completed informed consent and then confirmed eligibility. Subsequently, participants complete an audio computer-assisted self-interview (ACASI) survey using an iPad (Apple Inc). Survey items included questions pertaining to demographics, sexual behaviors, HIV risk behaviors, relationships, employment, income, substance use, and other structural variables such as homelessness, food insecurity, and health care use. Optional biologic samples were collected from participants who consented to them. These samples included a finger stick for rapid HIV testing using the Determine HIV-1/2 Ag/Ab Combo (Alere Inc), a urine sample for drug screening and chlamydia and gonorrhea testing, and anal and oral swabs for chlamydia and gonorrhea testing.

#### Intervention

Participants completed a 4-session intervention workshop adapted from the existing iFOUR program [41-43] and piloted in phase 2 of W2P. In phase 3, workshop sessions occurred over a 2-day period, with 2 sessions on each day. Session 1 focused on goal setting and identifying strengths; session 2, on communication, networking, and job searching; session 3, on balancing work with health and wellness; and session 4, on preparing job application materials and interview preparation. For a full description of the process for determining these intervention components, please refer to the W2P phase 1 protocol paper [71].

Workshop sessions were delivered by 2 facilitators in groups of 6 to 12 participants across the course of 2 days, with 2 sessions per day. The W2P Career Readiness Workbook was used as a guide for all workshop sessions and given to all study participants at the first session. Facilitators used an annotated W2P Facilitator Guide that provides detailed instruction on delivery of the intervention curriculum. During each session, facilitators completed a fidelity assessment to help ensure fidelity to the W2P Career Readiness Workbook, and after each session, they completed a workshop debriefing form to capture any workshop notes or comments.

#### Postintervention

Once participants completed the workshop sessions, they completed a postintervention ACASI survey using an iPad. Survey items included questions on workshop evaluation, job-seeking self-efficacy, and pre-exposure prophylaxis and HIV testing use.

#### 3-Month Follow-up

The final study visit occurred 3 months after the intervention was completed. During this visit, participants completed the baseline ACASI survey using an iPad and provided repeat biologic samples, if they consented to them.

### Outcomes

#### Primary Outcomes

The primary outcomes of this study are (1) information systems success model (ISSM) score, (2) workshop completion, (3) change in job-seeking self-efficacy scale score, and (4) change in protean career attitudes (PCA) scale score.

First, the ISSM will be used to assess for intervention acceptability and satisfaction. The 21-item scale yields a total score and measures 4 subdomains: information quality, handbook quality, perceived usefulness, and overall satisfaction. This scale has been adapted from Horvath et al [72].

Second, workshop completion will be used to assess intervention feasibility. Workshop or intervention completion is defined as having attended at least two of the 4 workshop sessions and is measured by tracking participant attendance.

Third, job-seeking self-efficacy is defined as one’s perceived ability and confidence to perform job search and application activities. The 12-item job-seeking self-efficacy scale by Barlow et al [73] yields a total score in which higher values indicate higher self-efficacy. Job-seeking self-efficacy was previously found to be associated with employment in a previous study of transgender women of color [61].

Fourth, PCAs are defined as self-direction in the pursuit of success in one’s work. Protean career attitudes have previously been found to be associated with positive career satisfaction and self-perceived success [74]. The validated 7-item scale by Porter et al [75] yields a total score and measures 2 subdomains: self-directed attitudes and values-driven attitudes.

#### Secondary Outcomes

Secondary outcomes include (1) change in self-reported hours worked per week, (2) change in self-reported sexual risk behaviors, (3) change in chlamydia test result, (4) change in gonorrhea test result, and (5) reactive HIV test.

First, hours worked per week was self-reported at baseline and at the 3-month follow-up visit. Change in hours worked per week from the baseline to the 3-month follow-up will be used to assess change in employment status.

Second, sexual risk behaviors are defined as self-reported engagement in the following behaviors during the previous 3 months [76]: (1) condomless anal intercourse (receptive or insertive) with cisgender male partner of unknown HIV status, (2) anal intercourse (receptive or insertive) with at least 3 cisgender males, (3) sex with cisgender male partner with an STI, (4) condomless anal intercourse (receptive or insertive) with HIV-positive cisgender male partner, (5) anal intercourse (receptive or insertive) with condom failure, and (6) transactional sex work involvement. The previous 3 months refers to the 3 months prior to the baseline visit for the first assessment and the 3 months prior to the 3-month follow-up visit for the second assessment. Change in sexual risk behaviors is defined as the change in self-reported behaviors from baseline to the 3-month follow-up.

Third, prevalence of chlamydia infections was assessed at baseline and 3-month follow-up using oral, anal, and urine samples. Each of the 3 tests yields a positive or negative result. Change in chlamydia test result is defined as the change from baseline to the 3-month follow-up. Oral, anal, and urine tests are treated as separate outcomes.

Fourth, prevalence of gonorrhea infections was assessed at baseline and 3-month follow-up using oral, anal, and urine samples. Each of the 3 tests yields a positive or negative result. Change in gonorrhea test result is defined as the change from baseline to the 3-month follow-up. Oral, anal, and urine tests are treated as separate outcomes.

Fifth, testing for reactive or nonreactive HIV was assessed at baseline and 3-month follow-up. The reactive HIV test outcome uses the 3-month follow-up result.

### Power

Given the exploratory nature of this study and limited access to this population, the analyses are not designed to have a specified level of statistical power. A repeated measures pretest-posttest design will be used to reduce the variability in the estimate of the treatment effect.

### Statistical Analysis

The analytic plan will estimate preliminary efficacy of the intervention by comparing preassessments and postassessments of employment and sexual risk behaviors. Descriptive statistics will be used to analyze the proportions and central tendencies for participant sociodemographic characteristics collected in the surveys. We will first generate frequencies, means, and other measures of central tendency as appropriate to describe our sample and outcomes at each of the 3 time points: baseline, postintervention, and 3-month follow-up.

All participants who were enrolled at baseline and completed the baseline ACASI will be included in the primary and secondary analyses as applicable. Analysis population participants will be included in all primary and secondary analyses for which their data for the specified outcome are not missing. Participants who did not attend any workshop sessions will not be included in analyses involving workshop evaluation. Primary analyses will assess intervention acceptability, satisfaction, and feasibility, as well as change in job-seeking self-efficacy and PCA score. Secondary analyses will evaluate the intervention by comparing preintervention and postintervention employment and sexual risk behaviors.

Changes in primary and secondary outcomes between baseline and 3-month follow-up will be assessed using paired 2-tailed *t* tests for continuous variables (eg, ISSM, job-seeking self-efficacy, and PCA scores) and the McNemar test for matched categorical variables (eg, STI results). We will use standard diagnostic tools to assess the appropriateness of the normality assumption and, if approximate normality of the residuals is not tenable, a nonparametric test for continuous paired data (ie, Wilcoxon signed rank test) will be used. All hypothesis testing will be performed at an α level of .1, given the exploratory nature of the study. To the extent that data allow, multivariable analyses will adjust for sociodemographic characteristics, workshop attendance, baseline employment status, and study completeness. Analytical models will include linear regression or generalized linear models for continuous outcomes and logistic regression for binary outcomes.

Analysis of the primary and secondary outcomes are described in detail within the statistical analysis plan, which will be accessible on ClinicalTrials.gov once study results have been entered.

## Results

Phase 3 of W2P research activities began in May 2019 and was completed in December 2019. Overall, 41 participants were enrolled in the community-based pilot.

## Discussion

Interventions that address the complex socioecological factors that make YMSM, YTW, and GNC youth of color vulnerable to HIV are necessary to curb the epidemic in this population. Given the role of social determinants of health in HIV infections experienced by these young people, interventions must explicitly address factors such as unemployment, homelessness, and survival sex work in order to maximize the impacts of individual-level behavior changes intended to mitigate vulnerability for adolescent and young adult HIV.

In the first 2 phases of W2P, the intervention was adapted from iFOUR, tailored to YMSM, YTW, and GNC youth of color, and then evaluated in a university setting. The goal of phase 3 of W2P is to pilot test this structural-level employment intervention in a community-based setting that serves the target population. Testing the intervention in a community setting is an opportunity to evaluate how recruitment, retention, and other outcomes are impacted by delivery in a venue more akin to where this intervention could eventually be used by nonresearchers. Given recent findings that identify the south side of Chicago as largely lacking services targeting LGBTQ communities’ specific needs [18], the selected site approximates well the most likely venue where this kind of intervention could be received by members of the target population. Further, by virtue of the study recruiting from and delivering the intervention in a community-based clinic that provides a range of supportive services, such as housing assistance, counseling, and HIV/STI testing, participants are in closer proximity to resources targeting a range of social determinants of health.

Given that the intervention was delivered to YMSM, YTW, and GNC youth of color in an urban LGBTQ community setting, deployment of the intervention in suburban or rural communities may require additional adaptation and refinement. Although implementing the employment intervention in an LGBTQ community–based setting offers an opportunity for direct recruitment and enrollment, the intervention may not reach YMSM, YTW, and GNC youth of color who are not connected with LGBTQ community and social services. Thus, additional approaches may be needed to reach the most vulnerable youth who are experiencing socioeconomic hardship.

If W2P demonstrates feasibility and acceptability when delivered in a community-based setting, we plan to test the efficacy in a multicity longitudinal trial across the ATN study sites. Phase 3 of W2P tested the intervention in one community venue. A scale-up of the project will allow the intervention to be tested in a range of community venues that may differ in ways that are relevant to outcomes, such as space, staff experience with intervention delivery, or availability of other resources. This will be an opportunity to further assess efficacy and identify implementation challenges and opportunities. If W2P demonstrates efficacy in the multicity trial, the intervention could be an asset for community organizations invested in addressing the role of employment in the HIV epidemic. Further, this intervention, which has been tailored to the target community and evaluated in relevant community-based settings, will provide YMSM, YTW, and GNC youth of color a way to gain employment skills needed to improve their economic situation and reduce HIV transmission.

## Abbreviations

ACASI: audio computer-assisted self-interview
ATN: Adolescent Medicine Trials Network for HIV/AIDS Interventions
CDC: Centers for Disease Control and Prevention
GNC: gender nonconforming
iFOUR: Increased Individual Income and Independence
ISSM: information systems success model
LGBTQ: lesbian, gay bisexual, transgender, and queer
MSM: men who have sex with men
PCA: protean career attitudes
STI: sexually transmitted infection
W2P: Work2Prevent
YMSM: young men who have sex with men
YTW: young transgender women
